# "Raising a Ghost It Were Better to Lay"

**Published:** 1917-11-10

**Authors:** C. O. Hawthorne


### THE EDITOR'S LETTER-BOX.

### "Raising a Ghost it were Better to Lay."

*To the Editor of* The Hospital.

Sir,—Your correspondent prudently avoids the greater part of my letter and thus achieves the brevity on which he prides himself. My own inclination is rather to compliment him on his discretion. Valour peeps out at the end of his letter, but soon retires itself. Otherwise my poor "periphrases" were to suffer "a good hard knock." To brevity, it seems, even boldness must give place. He now proposes a new issue, and one entirely different from that raised in his original article. There he affirmed that the B.M.A. shrank from making a claim on the War Office, while, in similar circumstances, it pressed a demand on the Pensions Committees; and he suggested that this difference of procedure was dictated by the conviction that the Pensions Committees would be more easy "to milk." In reply, I have shown that in a given instance —namely, that of the V.A.D. practitioners—the Association has not hesitated to apply its policy of State pay for State services to the War Office, even as in parallel circumstances it has applied this policy to the Pensions Committee. Further, I have explained that the non-action of the Association in the case of the visiting staffs of voluntary hospitals which receive wounded soldiers is compelled by the fact that the staffs themselves make no claim for payment. Where there are no claimants, surely no one can be blamed for failing to offer support. So far as I can follow his letter of November 3 the writer appears to believe that the V.A.D. claim, as supported by the Association, is made on the Pensions Committee. As a matter of fact, the claim, as I have already stated, is on the War Office, and anyone who does not know this has neglected to inform himself of one of the essential facts of the situation.

Your correspondent now proposes to inquire why some members of the profession ask for payment for work done for the State while others prefer to give gratuitous service, and he attempts to find in this difference a justification for his original article. The evasion is an ingenious one, but your readers will hardly be misled by it. His charge of inconsistency, of mean motives, and of cowardice was a charge directed against the B.M.A., and he cannot escape the responsibility of this by an endeavour to start a speculative inquiry why all members of the profession do not think alike on the question of State payments. He must be held to the specific indictment which he himself has framed. Until he faces this position his further flights do not concern me.—I am, Sir, yours faithfully, C. O. Hawthorne.

November 5, 1917.

